# Investigating the validity of the DN4 in a consecutive population of patients with chronic pain

**DOI:** 10.1371/journal.pone.0187961

**Published:** 2017-11-30

**Authors:** Hans Timmerman, Monique A. H. Steegers, Frank J. P. M. Huygen, Jelle J. Goeman, Nick T. van Dasselaar, Marcel J. Schenkels, Oliver H. G. Wilder-Smith, André P. Wolff, Kris C. P. Vissers

**Affiliations:** 1 Radboud university medical center, Department of Anesthesiology, Pain and Palliative Medicine, Nijmegen, the Netherlands; 2 ErasmusMC, Department of Anesthesiology, University Center of Pain Medicine, Rotterdam, the Netherlands; 3 Leiden University Medical Center, Department of Medical Statistics and Bioinformatics, Leiden, the Netherlands; 4 Reinier de Graaf Gasthuis, Department of Anesthesiology, Pain Medicine and Palliative Care, Delft, the Netherlands; 5 Bernhoven Ziekenhuis, department of Anesthesiology, Uden, the Netherlands; 6 Aalborg University, Center for Sensory-Motor Interaction, Aalborg, Denmark; 7 University of Groningen, University Medical Center Groningen, Department of Anesthesiology, Pain Center, Groningen, the Netherlands; University of Würzburg, GERMANY

## Abstract

Neuropathic pain is clinically described as pain caused by a lesion or disease of the somatosensory nervous system. The aim of this study was to assess the validity of the Dutch version of the DN4, in a cross-sectional multicentre design, as a screening tool for detecting a neuropathic pain component in a large consecutive, not pre-stratified on basis of the target outcome, population of patients with chronic pain. Patients’ pain was classified by two independent (pain-)physicians as the gold standard. The analysis was initially performed on the outcomes of those patients (n = 228 out of 291) in whom both physicians agreed in their pain classification. Compared to the gold standard the DN4 had a sensitivity of 75% and specificity of 76%. The DN4-symptoms (seven interview items) solely resulted in a sensitivity of 70% and a specificity of 67%. For the DN4-signs (three examination items) it was respectively 75% and 75%. In conclusion, because it seems that the DN4 helps to identify a neuropathic pain component in a consecutive population of patients with chronic pain in a moderate way, a comprehensive (physical-) examination by the physician is still obligate.

## Introduction

Neuropathic pain is described as pain caused by a lesion or disease of the somatosensory nervous system and requires a demonstrable lesion or a disease that satisfies established neurological diagnostic criteria[1]. Moreover, neuropathic pain is a clinical description and not a diagnosis[1]. In daily clinical practice it is to our opinion more appropriate to speak of a present neuropathic pain component (present NePC) or absent neuropathic pain component (absent NePC)[2, 3]. This because the pain experienced by the patient in the clinical context may be caused by both neuropathic- as well as nociceptive mechanisms (also known as ‘mixed pain’)[2, 4–6]. The main features of neuropathic pain components are, in clinical practice, the painful signs and symptoms in a region of altered sensations (numbness or increased sensitivity)[6]. The assessment of neuropathic pain is nowadays primarily based on history and physical examination including (bedside-)sensory testing[7–9] to assess patients’ pain.

Since current pharmacological treatment of patients with and without a NePC differs strongly, a correct pain classification is imperative[7, 10]. The availability of a simple and validated screening tool to determine the presence of NePC for clinical triage and epidemiological purposes can assist in detection of NePC[7, 8, 11–16]. This is especially true when this tool can be used by non pain specialists.

The original French validation study of the ‘Douleur Neuropathique en 4 Questions’ (DN4)[17] was performed in patients with neuropathic pain resulting from, for example, nerve trauma or post herpetic neuralgia. Patients with non-neuropathic pain were, amongst other diagnoses, suffering from osteoarthritis. All included patients had pain of at least a moderate severity (≥ 40 on a 100mm visual analogue scale). Pain classification in this study was based on medical history, physical examination, electromyography and/or imaging by two independently working physicians. DN4 application resulted in a sensitivity of 83% and a specificity of 90%[17]. As indicated in a systematic review by Mathieson et al[16] the classification of a NePC may differ between clinicians and may be more difficult when there are patients included with mixed pain and with all levels of pain. This reflects the patient population in a daily clinical practice, but might have an influence on the validity. Moreover, the accuracy of screening tools is dependent on the standardization of the assessment strategy[18]. Translation/ cross-cultural adaptation and/or validation of the DN4 was performed in more than 75 languages[19–31].

The neuropathic pain special interest group (NeuPSIG) grading system[32] is developed by Treede et al in 2008 and updated in 2016[33]. It is a system to help the clinician to determine the certainty of the pain classification for the existence of a NePC in an individual patient: non-neuropathic pain; possible, probable or definite neuropathic pain. The grading system is suggested to be helpful in the assessment of the pain classification in clinical practice[34–38].

The aim of this study was to assess the validity and reliability of the DN4 as a screening tool for use in daily outpatient practices to detect a NePC in a, not pre-stratified on the target outcome, consecutive patient population having chronic pain syndromes due to low back and leg pain (LBLP), neck-shoulder-arm-pain (NSAP) or pain of suspected neuropathic origin (PSNO).

## Methods

This validity and reliability study had a cross-sectional, longitudinal, research design with a 2-weeks and 3-months follow-up period. Comparisons were made between the DN4 (as a whole and for the symptom questions and signs tests separately) and the classification of patients’ pain by two, independently working, physicians (the gold standard) as well as with the grading system.

The study was approved by the Committee on Research Involving Human Subjects region Arnhem-Nijmegen, Nijmegen, the Netherlands, (dossier number: 2008/348; NL 25343.091.08) which counts for participation of the Dutch academic pain centers (Radboud University Medical Center, Nijmegen; Utrecht University Medical Center, Utrecht; Erasmus Medical Center, Rotterdam), Dutch non-academic pain centers (Bernhoven Ziekenhuis, Oss; St.Anna Ziekenhuis, Geldrop) and a Dutch non-academic department of neurology (Rijnstate Ziekenhuis, Arnhem). Participation of Dutch non-academic pain center in Delft, the Netherlands (Reinier de Graaf gasthuis) was approved by Medisch Ethische Toestings Commissie Zuidwest Holland (dossier number: 10–145). The study protocol was registered in the Dutch National Trial Register (NTR3030).

We used the same methodology as in the published protocol [39] and as employed in a simultaneous study regarding the validity of the Pain*DETECT* (Timmerman et.al / Under review by BMC Neurology).

### Participants

Consecutive patients (first time visitors of the participating centers) without pre-stratification based on the target outcome[40] were included in the study between October 2009 until July 2013. Patients were asked to participate by their doctor. Each patient signed informed consent before participation in the study.

At that time, there was only a rough diagnosis: LBLP, NSAP or PSNO. Inclusion criteria: Male and female adult patients (≥18 years of age) with chronic (≥3 months) LBLP or NSAP radiating into respectively leg(s) or arm(s) or patients with chronic pain due to a PSNO (pain associated with a lesion or disease of the peripheral somatosensory system). Exclusion criteria: Patients diagnosed with malignancy; compression fractures; patients with diffuse pains (such as fibromyalgia or ankylosing spondylitis); severe mental illness; chronic alcoholism or substance abuse; inability to fill in the questionnaire adequately or incapable of understanding the Dutch language.

### Physicians

The physicians (pain specialists, pain specialist fellows or neurologists always operating in differently composed pairs) participating in this study were not selected on basis of age, experience as a physician or any other criteria. Classification of patients’ pain was based on the NeuPSIG guidelines on neuropathic pain assessment[7] and recorded as absent NePC or present NePC. Pain classification was performed consecutively on the same patient by two physicians and categorized afterwards in three groups: absent NePC, present NePC or ‘undetermined’ (i.e. the pain classification of the two physicians was not the same). A full medical history and clinical examination including sensory bedside examination (touch, pinprick, pressure, cold, heath and temporal summation) was taken[7, 8, 39, 41, 42] and was considered to be the gold standard when assessed by two physicians. The NeuPSIG grading system[32, 33] was used as a secondary comparison with the outcome of the DN4 and was assessed by both the physicians separately. The outcomes “probable” and “definite” were regarded as present NePC. “unlikely” and “possible” as absent NePC[38, 43, 44]. The physicians worked independently of each other and were blinded to the pain classification of the other physician. Each physician was allowed to perform the clinical examination in the way he or she is used to do but were supported by a standardized assessment form[39]. In this form, the pain score, a body map to indicate the localization of patients pain, the sensory examination and the four questions of the grading system had to be filled in by the physician. The participating physicians were trained in a standardized way (presentation about the study and the outcome parameters and a practical training on how to use the (measurement) instruments), by the investigator (HT) or by a designated person on location before participation in the study. Practical training was focused on the classification of NePC, the assessment of the grading system, the performance of bed-side examination tests and the performance and assessment of the examination items of the DN4.

In this study, 62 physicians (pain specialist, pain specialist-fellow or neurologist) participated. The physicians who were classifying patients’ pain at the first session were called ‘Physicians A’. The physicians who performed the classification at the second session, were called ‘Physicians B’.

### Measurements

#### Douleur Neuropathique en 4 questions (DN4)

The DN4 [17, 20, 25] (Pfizer bv. Capelle a/d IJssel, the Netherlands) consists of 10 items in total and is developed to screen for symptoms and signs of neuropathic pain resulting in a yes/no answer for the presence of neuropathic pain. This instrument is divided into two questions (seven answers, DN4-symptoms: score range 0–7) and two physical examination tests (three answers, DN4-signs: score range 0–3). The examination items of the DN4 regarding the signs (hypoesthesia to touch, hypoesthesia to prick and brushing) were incorporated in the sensory examination part of the standardized assessment form and were carried out according the original publication by Bouhassira et al [17]. This assessment form was filled in by both physicians separately. The seven symptom items are consisting of characteristics (Burning, painful cold, electric shocks) and symptoms (Tingling, pins and needles, numbness, and itching). The patient completed the DN4-symptoms directly after the clinical assessments by the physicians but without interference. The researcher (HT) or a nurse was available for help in person or via telephone when it was not clear fort the patient how to fill in the questionnaires.

The items of the DN4 are scored based on a yes (1 point) /no (0 points) answer. This leads to a score range of 0–10 when the symptoms (range 0–7 points) as well as the signs (range 0–3 points) items are included. Values in the DN4 who were not filled in were considered as ‘no’ (0 points). However, in the reliability analysis these data were not incorporated.

#### Patient global impression of change (PGIC)

The Patients Global Impression of Change (PGIC)[11, 45–47] was used to assess the change of pain complaints, based on the patients’ own impression of change over time, during the follow-up period (7-points scale: Very much improved-very much worse). Follow-up took place two weeks and three months after the initial visit. To compare the outcome of the DN4 in the follow-up period the pain complaints as addressed by the patient had to be unchanged.

### Time-line

All baseline measurements (the assessment by the physicians, the grading system by both physicians as well as filling in the questionnaires by the patient) took place on preferably the same day. The PGIC [45–47] and the DN4-symptoms (sensory testing for the DN4-signs was not performed) were sent to the patient after two weeks and three months with instructions how to fill them in by mail. Also for the follow-up measurements help was available in person or via telephone when it was not clear how to fill in the questionnaires.

### Data

All data was collected on paper and stored by Radboudumc, Nijmegen, The Netherlands. Data management and monitoring were performed within MACRO (MACRO, version 4.1.1.3720, Infermed, London, United Kingdom). Data analysis and statistics was performed by use of Statistical Package for the Social Sciences (IBM SPSS statistics 22, SPSS Inc., Chicago, Illinois, USA).

### Statistical analysis

According to the power-calculation in the protocol 132 patients with LBLP, NSAP or PSNO were needed such that the sample size contains adequate numbers of cases and controls[39]. Qualitative variables are presented as frequencies and percentages. The quantitative variables are presented as mean and standard deviation (SD) or as median and inter quartile range (IQR).

The agreement between any of the two combinations of the two observers (pain classification by the physician and the outcome of the grading system) to establish a present NePC or absent NePC, and of the DN4 (DN4 / DN4-symptoms / DN4-signs outcome) was evaluated by use of Cohen’s kappa (K), prevalence index (Pi) and percentage of pair wise agreement (PA). The categorization of the kappa values are, according to the categorization of observer agreement by Landis and Koch[48], none beyond chance (K≤0.00); slight (K = 0.01–0.20); fair (K = 0.21–0.40); moderate (K = 0.41–0.60); substantial (K = 0.61–0.80) and (almost) perfect agreement (K = 0.81–1.00). A Κ ≥ 0.40 and a PA ≥ 70% is considered indicative of interobserver reliability acceptable for use in clinical practice[48]. Moreover, also the interobserver reliability of the examination items in the DN4-signs were tested.

Based on the classifications of the two physicians, all patients were categorized as absent NePC, present NePC or ‘undetermined’ (i.e. the pain classification of the two physicians was not the same).

Statistical significant differences between absent NePC and present NePC were determined by use of students t-test (Interval scales), Mann-Whitney U-test groups (ordinal scales) or via Chi^2^-test (nominal scale). The statistical significant differences between present NePC, absent NePC and the Undetermined group was assessed by use of One-way ANOVA (with additional Tukey’s studentized range post-hoc test) or Kruskal-Wallis test. Chi^2^ test was also used to analyze the nominal outcome scale of the DN4 regarding the three groups.

A factor analysis was used to study the structure of the DN4 in such a way that variables that were thought to reflect a smaller number of underlying variables were observed. This method was performed for all three versions of the DN4 (DN4; DN4-symptoms and DN4-signs). Principal axis factoring was used as the extraction method. The varimax rotation with Kaiser normalization was used. Extraction of the factors was based on Eigenvalues being greater than 1.0. Cronbach’s alpha was used to calculate the internal consistency of the factors constructed. The results are only shown for the Physicians A (the assessment of the patient by the first physician). The outcomes by the Physicians B (the assessment of the patient by the second physician) are shown in S1 Table. However, the conclusions, which are drawn, are identical for physicians A and for physicians B.

A receiver operating characteristic (ROC) curve was calculated for the DN4 and the DN4 signs by both the physicians A and B and for the DN4-symptoms as filled in by the patient. The area under the curve (AUC) with 95% confidence interval was presented to indicate the discriminatory power of the DN4 to discriminate patients by present NePC or absent NePC. This dichotomy was based on the physicians’ assessment outcome or based on the grading system outcome, respectively. The theoretical maximum of the AUC is 100%, indicating a perfect discrimination and 50% is equal to tossing a coin. An AUC between 0.9 and 1 is considered to be excellent, an AUC between 0.8 and 0.9 is good and between 0.7 and 0.8 is fair. An AUC between 0.6 and 0.7 is considered to be poor. Between 0.5 and 0.6 the AUC is considered to be failed[49–52]. The optimal cut-off point of the DN4 was calculated under the condition of equal-costs of misclassification using the Youden-index. Sensitivity, specificity, positive and negative predictive values and the likelihood ratio in the population in this study was calculated at this cut-off point. The outcome results were averaged between both physicians and the 95% confidence intervals were noted with respect to the lowest and highest level.

Clinimetrics of the DN4 based on both the physicians assessment and/or both the grading system outcome were assessed for the DN4, the DN4-symptoms and for the DN4-signs items. A screening tool for the presence of a NePC is considered valid if it has a high sensitivity, specificity, high positive predictive value and a high positive likelihood ratio [53].

Intraclass correlation (ICC) was used to assess reproducibility (‘test-retest reliability’) of the DN4-symptoms between the predetermined time points (baseline versus two weeks & baseline versus three months). Based on the guidelines by Cicchetti et al.[54, 55] an ICC <0.40 indicates poor level of clinical significance. The level is fair when the ICC is between 0.40 and 0.59, good between 060 and 0.74 and excellent when the ICC is between 0.75 and 1.00. To assess the test-retest reliability patients’ pain should not have changed (outcome based on the PGIC) because otherwise the ICC would not reflect the consistency of the DN4. Test-retest reliability was assessed for those questionnaires returned within 7–21 days for the two weeks test-retest reliability and 60–120 days for the three months test—retest reliability. The ICC and responsiveness of the DN4-symptoms was assessed at each point of measurement.

Two-tailed p-value below 0.05 was considered statistically significant.

## Results

### Patients

**Fig 1:**
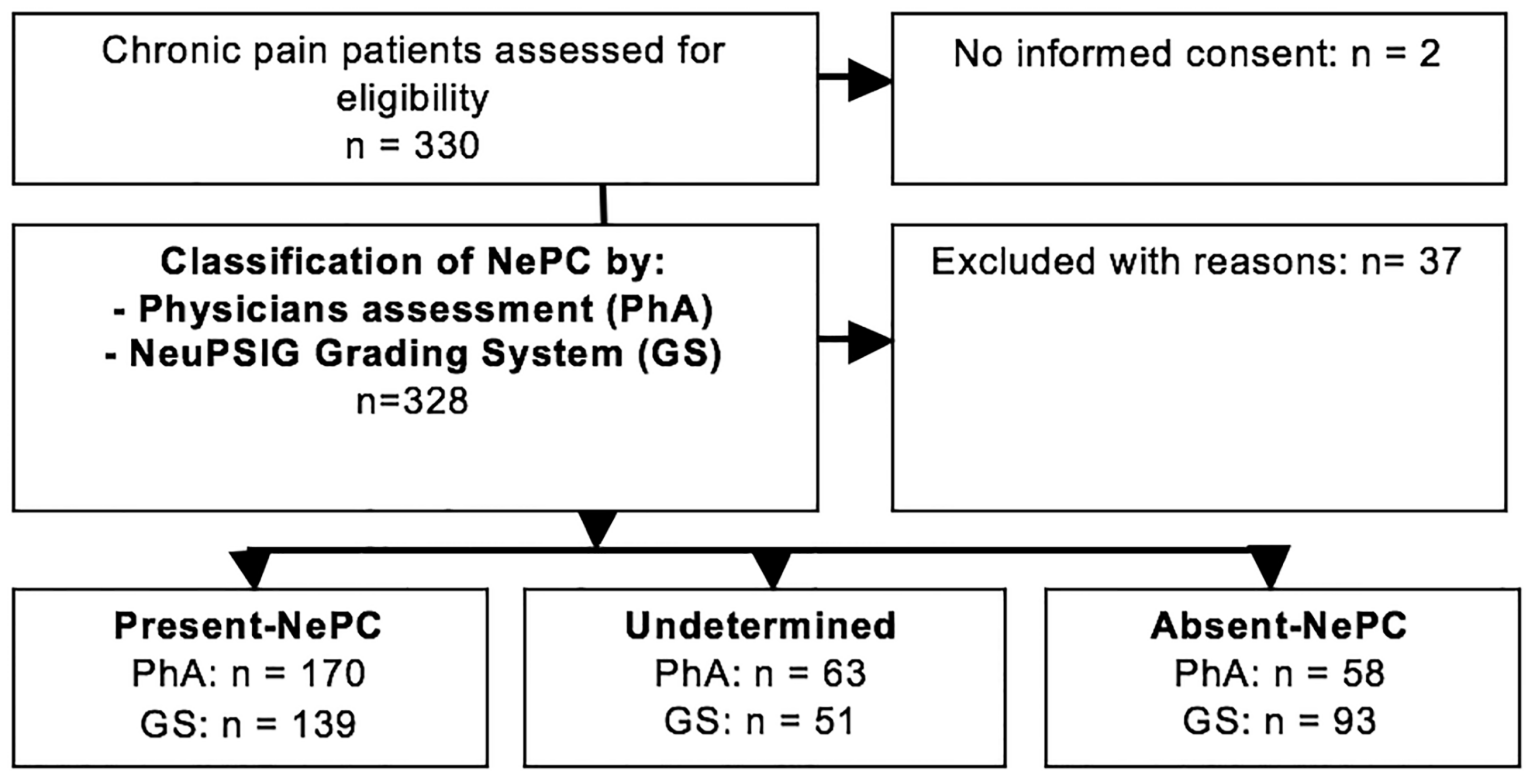
Flow diagram for the outcome of the physicians assessment and the NeuPSIG grading system. Present NePC: present neuropathic pain component; Undetermined: Both physicians disagree with each other about the existence of a neuropathic pain component; Absent NePC: absent neuropathic pain component; n = total number of patients in analysis PhA: Physicians assessment; GS: Neuropathic pain special interest group grading system (missing pain classification based on the grading system: n = 8).

In this study 330 consecutive patients were assessed for eligibility (Fig 1). Of these, 291 participated in the study between October 2009 and July 2013. Two patients did not give their informed consent. Exclusion (n = 37) was because of not fulfilling the in- and exclusion criteria (n = 13): patients with LBLP or NSAP without radiating pain: n = 1; patients with less than 3 months pain complaints: n = 2; patients with pain with an oncological cause: n = 2; patients with painful syndromes of unknown origin or associated with diffuse pains: n = 7; patients with severe mental illness: n = 1; missing baseline measurements due to not returning questionnaires by the patient: n = 16; missing pain classification based on the grading system by one physician (n = 5) or both the physicians (n = 3). 132 patients had LBLP with radiation in one or two legs (45.4%), 51 NSAP with radiation in one or both arms (17.5%) and 108 patients (37.1%) had PSNO: 86 patients with pain after treatment for breast cancer (surgery and chemotherapy and/or radiation therapy and/or hormonal therapy). Twenty-two patients had pain for various reasons: peripheral nerve damage (n = 12), radicular pain (n = 3), polyneuropathy (n = 3), CRPS (n = 2) and post stroke pain (n = 2). The gold standard for presence of the NePC in this study was the concordant clinical opinion of both physicians. After pain classification by two physicians, 170 patients were classified as present NePC, 58 as absent NePC and in 63 patients the two physicians made a different pain classification: ‘undetermined’. Using the grading system, 139 patients were assigned as having a present NePC, 93 patients as absent NePC and 51 patients were assigned as undetermined. The DN4 was full filled by the patients at a median of one day (IQR 0–5 days) following the assessments by the physicians.

Clinical and social-demographic details of the 291 patients were analyzed based on their pain classification. No statistically significant differences were found between present NePC and absent NePC for gender, age, height, weight, BMI, medication and duration of pain. Also no statistically significant difference was observed between absent NePC and present NePC regarding current- worst and average pain (Table 1).

**Table 1:** Clinical and socio-demographic characteristics of the patients related to physicians agreement for the existence of a neuropathic pain component.

	NePC	Absent		Present			Undetermined		
		N		N		*P*	N		*P*
**Total number of patients**		58		170			63		
**Gender**						*0*.*163*^c^			*0*.*164*^c^
	Male		25 (43%)		56 (33%)			17 (27%)	
	Female		33 (57%)		114 (67%)			46 (73%)	
**Age (Years)** ^#^		58	55 ± 12	170	56 ± 11	*0*.*594*^a^	63	58 ± 13	*0*.*522*^d^
**Height (cm)** ^#^		55	172 ± 9	164	172 ± 8	*0*.*845*^a^	6	170 ± 9	*0*.*250*^d^
**Weight (kg)** ^#^		55	84 ± 2	167	80 ±17	*0*.*382*^a^	6	80 ± 16	*0*.*461*^d^
**BMI (kg/m**^**2**^**)** ^#^		54	28 ± 8	164	27 ±5	*0*.*436*^a^	6	27 ± 5	*0*.*593*^d^
**Medication use**^^^		55	56.9%	168	66.1%	*0*.*414*^c^	61	57.4%	*0*.*423*^c^
**Duration of pain (months)** ^#^		57	72 ± 90	169	60 ± 76	*0*.*327*^a^	62	49 ± 46	*0*.*247*^d^
Pain* **(NRS; 0–10)**									
Current pain		57	5 (3–7)	167	6 (3–7)	*0*.*577*^b^	6	4 (1–7)	*0*.*084*^e^
Worst pain during the past four weeks		57	8 (5–9)	167	8 (7–9)	*0*.*371*^b^	6	7 (5–8)	*0*.*053*^e^
Average pain during the past four weeks		57	6 (3.5–7)	167	6 (5–8)	*0*.*233*^b^	6	6 (3–7)	***0*.*018***^e^

Classification for the existence of NePC is based on physicians assessment of the patients. NePC: neuropathic pain component; Absent: NePC is absent; Present: NePC is present; Undetermined: both physicians disagree with each other about the existence of a neuropathic pain component; N: total number of patients in analysis; n: number of patients;

^percentage;

^#^Standard deviation;

* Inter quartile range. A: physicians A; B: Physicians B; P value for significant difference between groups (P ≤ 0.05) by use of different analyse methods:

^a^: Students t-test;

^b^: Mann-Whitney U test;

^c^: Chi-square;

^d^: One-Way ANOVA;

^e^: Kruskal-Wallis test.

### Reliability

The proportion of agreement after chance agreement is removed (Cohen’s Kappa, K) for the classification of patients’ pain (absent NePC or present NePC) by the physicians was 0.49 (moderate), with a PA of 78.4% (Pi = 0.38; n = 291). For the classification of patients’ pain on basis of the grading system K was 0.63 (good) and PA was 82% (Pi = 0.16; n = 283). The outcome of K and PA regarding the DN4 compared to the outcome of the assessment by physicians A was respectively 0.34 (fair) and 69.8% (Pi = 0.33; n = 275). Compared to the outcome of the assessment by physicians B it was 0.33 (fair) and 69.2% (Pi = 0.30; n = 263). Comparing the outcome of the DN4 to the outcome of the grading system, it was 0.35 (fair) and 69.1%(Pi = 0.22; n = 272) for physicians A, and 0.32 (fair) and 67.3%(Pi = 0.19; n = 260) for physicians B (Table 2). The interobserver reliability for ‘hypoesthesia to touch’ as well as for ‘brushing’ was respectively K = 0.59 (moderate) (PA = 79.7%) and K = 0.53 (moderate)(PA = 76.6%). The interobserver reliability for ‘hypoesthesia to prick’ was K = 0.21 (fair); PA = 87% (Table 3).

**Table 2:** The kappa coefficient between the classification on basis of the assessment by the physicians, the grading systems, the DN4 and the kappa coefficient between both physicians regarding the DN4-signs.

		**Classification physician B**	**Grading A**	**Grading B**	**DN4 A**	**DN4 B**	**DN4 Symptoms**	**DN4-Signs A**	**DN4-Signs B**
**Classification physician A**	n	**291**	**286**	288	275	263	288	279	266
	*K*	**0.49**	**0.48**	0.32	0.34	0.34	0.32	0.37	0.26
	PA	**78.4**	**76.2**	67.4	69.8	70.0	67.4	70.3	64.4
	P*i*	**0.38**	**0.32**	0.26	0.33	0.31	0.26	0.30	0.30
**Classification physician B**	n		286	**288**	275	263	288	279	266
	*K*		0.38	**0.48**	0.33	0.33	0.21	0.39	0.37
	PA		71.0	**75.0**	69.1	69.2	62.8	71.0	70.7
	P*i*		0.28	**0.22**	0.29	0.30	0.25	0.26	0.28
**Grading A**	n			**283**	272	259	283	**276**	262
	*K*			**0.63**	0.35	0.31	0.14	**0.54**	0.31
	PA			**82.0**	69.1	67.2	58.6	**77.5**	67.2
	P*i*			**0.16**	0.22	0.23	0.19	**0.19**	0.21
**Grading B**	n				272	260	285	**276**	**263**
	*K*				0.29	0.32	0.13	**0.53**	**0.45**
	PA				65.4	67.3	57.2	**76.8**	**73.4**
	P*i*				0.18	0.19	0.14	**0.14**	**0.16**
**DN4 A**	n					**257**	**275**	**275**	257
	*K*					**0.76**	**0.62**	**0.52**	0.29
	PA					**88.7**	**81.8**	**76.7**	65.8
	P*i*					**0.21**	**0.19**	**0.17**	0.19
**DN4 B**	n						**263**	**257**	**263**
	*K*						**0.65**	**0.40**	**0.45**
	PA						**82.9**	**71.2**	**73.4**
	P*i*						**0.18**	**0.21**	**0.17**
**DN4 symptoms**	n							276	263
	*K*							0.15	0.10
	PA							58.7	56.3
	P*i*							0.17	0.16
**DN4-Signs A**	n								**260**
	*K*								**0.55**
	PA								**78.4**
	P*i*								**0.18**

n = total number of patients in the analysis; K = Cohen’s kappa value; PA (%) = percentage of agreement between two outcome variables; Pi = Prevalence index

**Table 3:** The kappa coefficient between both physicians regarding the DN4-signs.

		**Hypoesthesia to touch** DN4-signs B	**Hypoesthesia to prick** DN4-signs B	**Brushing** DN4-signs B
**Hypoesthesia to touch** DN4-signs A	n	**222**		
	*K*	**0.59**		
	PA	**79.7**		
	P*i*	**0.10**		
**Hypoesthesia to prick** DN4-signs A	n		244	
	*K*		0.21	
	PA		87.3	
	P*i*		-0.82	
**Brushing** DN4-signs A	n			**222**
	*K*			**0.53**
	PA			**76.6**
	P*i*			**0.11**

n = total number of patients in the analysis; K = Cohen’s kappa value; PA (%) = percentage of agreement between two outcome variables; Pi = Prevalence index

In 253 patients all the six outcome variables (two times the physicians’ assessment, two times the grading system and The DN4 by physician A and DN4 by physician B was available. In 83 patients (32.8%), the pain was classified as present NePC in all outcomes and in 22 patients (8.7%) it was six times negative, indicating absent NePC, so the agreement on all the six measures was 41.5% (the percentage of agreement based on both the gold standards and both the grading systems only was 56.9%).

### Factor analysis

Table 4 shows the loading factor of the items of the DN4 according to the rotated component matrix factor analysis with Kaiser normalization. The analysis was performed by use of the 10 questions in the DN4 and revealed a 4-factor solution explaining 59.3% of the variance for the first physicians’ assessment (physicians A): Factor 1 included two items (hypoesthesia to touch, brushing) indicating that there was an inter-relation between those items (Cronbach’s α: 0.87). Factor 2 included three items (painful cold, tingling, hypoesthesia to prick) (Cronbach’s α: 0.37). Factor 3, consisted of four items (burning, electric shocks, pins and needles, numbness); Cronbach’s α: 0.51). Factor 4 consisted of one item (itching) (Table 4). In the S1 Table we provided the factor analysis for both the physicians assessments (A & B), the DN4 symptoms solely and the DN4signs for both physicians’ assessments (A & B). Internal consistency of all the components of the DN4 for the physicians A at baseline was assessed via Cronbach’s α: 0.57; for the physicians B it was 0.55. Cronbach’s α for DN4-symptoms was 0.52. Cronbach’s α for the DN4-signs for A and B were respectively 0.68 and 0.66.

**Table 4:** Loading factors of the items of the DN4 according to the rotated component matrix factor analysis.

DN4	Component (Physicians A)			
	1	2	3	4
**Burning**	0.25	0.28	**0.29**	0.15
**Painful cold**		**0.62**		
**Electric shocks**			**0.72**	
**Tingling**		**0.68**		
**Pins and needles**		0.35	**0.45**	0.27
**Numbness**			**0.71**	
**Itching**				**0.86**
**Hypoesthesia to touch**	**0.87**			
**Hypoesthesia to prick**	0.38	**0.63**		
**Brushing**	**0.90**			
***Cronbach*’*s alpha***	*0*.*81*	*0*.*37*	*0*.*51*	

Loading factors < 0.25 are omitted to improve readability

### Items of the DN4

The DN4-symptoms (pain descriptors) burning, electric shocks, tingling, pins and needles, and numbness were statistically significant associated (Chi^2^) with the classification by the physicians (absent NePC, present NePC or undetermined), p<0.05. The descriptors ‘painful cold’ (p = 0.210) and ‘itching’ (p = 0.409) were not associated with the outcome of the classification. The DN4-signs (examination items) hypoesthesia to touch, pricking and brushing were statistically significant associated (Chi^2^) with the classification by the physicians (absent NePC, present NePC or undetermined), p<0.05.

The median of the total sum score of the DN4 for patients classified as absent NePC was 2, the median for the DN4-symptoms items was 2 and for the DN4-signs items the median was 0; for patients classified as present NePC it was at median 5, 3 and 2, respectively. As calculated based on the Kruskal-Wallis test there was for the sum scores of the DN4, the DN4-symptoms items and the DN4-signs items a statistical significant difference between absent NePC and present NePC (P<0.001), between present NePC and undetermined (P<0.001) and between absent NePC and undetermined (P<0.001). In Table 5 the outcomes for all individual items and the three DN4 scales (for physicians A as well as for physicians B) are presented according to the pain classification by the physicians (Table 5).

**Table 5:** The median (IQR) and percentages of the items of the DN4 by physicians agreement of a NePC.

NePC		Absent		Present			Undetermined		
		N		N		*P*	N		*P*
**Total number of patient**		58		170			63		
**DN4-Symptoms**^^^	Burning	56	12 (21%)	161	77 (48%)	***0*.*001***^a^	57	22 (39%)	***0*.*002***^a^
	Painful Cold	54	6 (11%)	154	34 (22%)	*0*.*078*^a^	53	11 (21%)	*0*.*210*^a^
	Electric Shocks	55	18 (33%)	162	87 (54%)	***0*.*007***^a^	55	19 (35%)	***0*.*005***^a^
	Tingling	55	29 (53%)	160	110 (69%)	***0*.*032***^a^	57	28 (49%)	***0*.*011***^a^
	Pins and Needles	52	19 (37%)	157	101 (60%)	***0*.*000***^a^	58	27 (47%)	***0*.*001***^a^
	Numbness	54	29 (54%)	165	131 (79%)	***0*.*000***^a^	59	42 (71%)	***0*.*001***^a^
	Itching	51	10 (20%)	149	25 (17%)	***0*.*646***^a^	56	14 (25%)	***0*.*409***^a^
**DN4-signs**^^^	Hypoesthesia to touch A	42	9 (21%)	153	102 (67%)	***0*.*000***^a^	60	16 (27%)	***0*.*000***^a^
	B	41	11 (27%)	151	101 (67%)	***0*.*000***^a^	49	18 (37%)	***0*.*000***^a^
	Hypoesthesia to prick A	47	0 (0%)	162	20 (12%)	***0*.*011***^a^	58	3 (5%)	***0*.*017***^a^
	B	48	0 (0%)	159	21 (12%)	***0*.*008***^a^	53	1 (2%)	***0*.*002***^a^
	Brushing A	43	9 (21%)	157	110 (70%)	***0*.*000***^a^	55	14 (25%)	***0*.*000***^a^
	B	43	13 (30%)	151	99 (66%)	***0*.*000***^a^	52	19 (37%)	***0*.*000***^a^
Total sum score DN4 A* (0–10)		47	2 (1–3)	166	5 (3–6)	***0*.*000***^b^	62	3 (2–4)	***0*.*000***^c^
Total sum score DN4 B* (0–10)		48	2 (2–3,75)	159	5 (3–6)	***0*.*000***^b^	56	3 (2–4.75)	***0*.*000***^c^
Total sum score DN4 symptoms* (0–7)		57	2 (1–3)	168	3 (2–5)	***0*.*000***^b^	63	2 (2–4)	***0*.*000***^c^
Total sum score DN4 signs A* (0–3)		49	0 (0–0)	168	2 (1–2)	***0*.*000***^b^	62	0 (0–1)	***0*.*000***^c^
Total sum score DN4 signs B* (0–3)		49	0 (0–1)	161	2 (0–2)	***0*.*000***^b^	56	0 (0–2)	***0*.*000***^c^

Classification for the existence of NePC is based on physicians assessment of the patients. NePC: neuropathic pain component; Absent: NePC is absent; Present: NePC is present; Undetermined: both physicians disagree with each other about the existence of a neuropathic pain component; N: total number of patients in analysis; n: number of patients;

^ percentage;

* Inter quartile range. A: physicians A; B: Physicians B; P value for significant difference between groups (P ≤ 0.05) by use of different analyse methods:

^a^: Chi-Squared;

^b^: Mann-Whitney U test;

^c^: Kruskal-Wallis test.

### Validity

**Fig 2:**
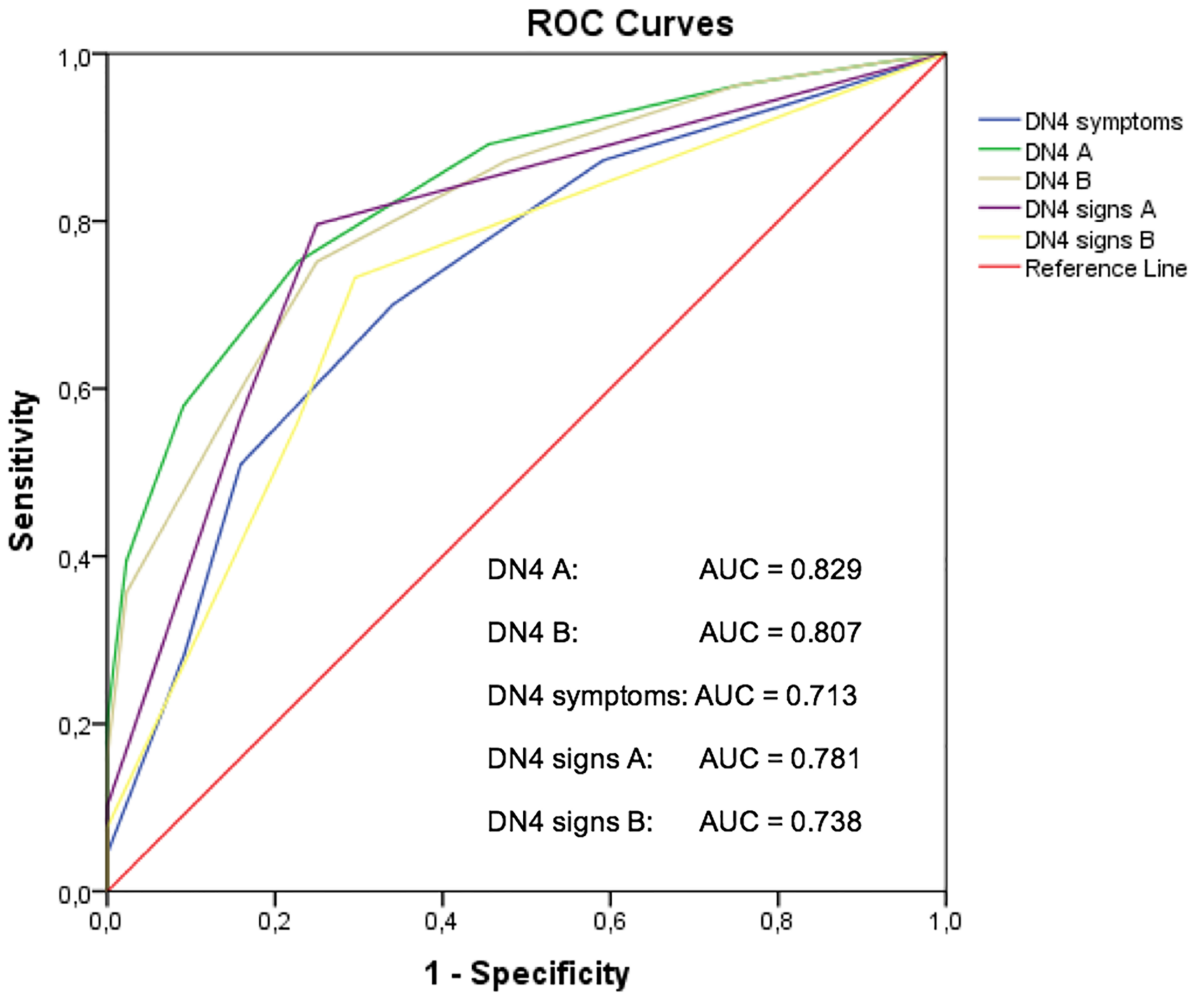
The ROC curve of the DN4, DN4 symptoms and the DN4 signs to the probability of the presence of NePC as classified based on the assessment by the physicians (A and B). DN4: Doleur Neuropathique en 4 questions; DN4-symptoms: the items filled in by the patient; DN4 A: DN4-symptoms filled in by the patient and DN4-signs as asssessed by physicians A; DN4 B: DN4-symptoms filled in by the patient and DN4-signs as asssessed by physicians B; DN4 signs A: DN4-signs as asssessed by physicians A; DN4 signs B: DN4-signs as asssessed by physicians B.

We constructed ROC-curves for the DN4, the DN4-symptoms and the DN4-signs with respect to the classification by physician A or B and according to the neuropathic pain grading system by physician A or B and all the combinations (Concordant assessment by physicians A and B together, concordant grading system by Physicians A and B together and concordant grading system for Physicians A and B together with the concordant grading system by physicians A and B). This because of the chosen gold standard and the grading system in which patients were classified by two different physicians. This might have lead to differences in the outcomes relative to the individual outcome by the physician. In Fig 2 the ROC-curve is displayed for the DN4 (physicians A and physicians B), DN4-symptoms and the DN4-signs (physicians A and physicians B) (Fig 2).

Based on the gold standard the sensitivity of the DN4 was on average (at maximal Youden-index, cut off point: 4/10) 75% (95% CI 0.68–0.81), specificity 76% (95% CI 0.61–0.86), positive predictive value 92% and the positive likelihood ratio was 3.09 (95% CI 1.82–5.39) (Table 5; S2 Table). For patients with LBLP the sensitivity was on average 75% and specificity was on average 81%. For patients with NSAP the averaged sensitivity was 73% and the specificity was on average 72%. For patients with pain due to a PSNO it was respectively, on average, 70% and 78%. The sensitivity of the DN4-symptoms was, in respect to the gold standard, 70% (95% CI 0.63–0.77) and the specificity was 67% (95% CI 0.54–0.78) (at maximal Youden-index, cut off point 3/7). Analysis of the DN4-signs solely resulted in an average sensitivity of 75% (95% CI 0.66–0.82) and an average specificity of 75% (95% CI 0.58–0.87) (at maximal Youden-index, cut off point 1/3). With the outcome based on the grading system the sensitivity was on average 76% (95% CI 0.68–0.82) and the specificity was 64% (95% CI 0.51–0.74) (at maximal Youden-index, cut off point 4/10). (Table 6; S2 Table).

**Table 6:** The area under the curve and the sensitivity / specificity at the optimal cut-off point of the DN4 under the condition of equal costs of misclassification to classify a neuropathic pain component by the classification and the grading system of the physicians.

	Present NePC	Absent NePC	AUC	(95%CI)	Youden index	Cut-off	Sens %	95% CI	Spec %	95% CI	PPV %	NPV	PLR	95% CI
**Classification A = B**														
**DN4 A**	**166**	**47**	**0.829**	**0.767–0.890**	**0.513**	**4**	**75**	**0.676–0.807**	**77**	**0.628–0.864**	**92**	**46**	**3.19**	**1.889–5.394**
LBLP	72	26	0.823	0.738–0.90	0.544	4	74	0.624–0.824	81	0.621–0.915	91	53	3.83	1.72–8.517
NSAP	23	10	0.763	0.576–0.950	0.439	3	74	0.535–0.875	70	0.397–0.892	85	54	2.46	0.927–6.547
PSNO	71	11	0.836	0.713–0.959	0.543	5	63	0.518–0.736	91	0.623–0.984	98	28	6.97	1.067–45.558
**DN4 B**	**159**	**48**	**0.807**	**0.742–0.872**	**0.498**	**4**	**75**	**0.678–0.81**	**75**	**0.612–0.851**	**91**	**47**	**2.99**	**1.819–4.927**
LBLP	67	26	0.821	0.736–0.906	0.554	4	75	0.631–0.835	81	0.621–0.915	91	55	3.88	1.744–8.637
NSP	21	11	0.725	0.529–0.921	0.442	4	71	0.5–0.862	73	0.434–0.903	83	57	2.62	0.961–7.135
PSNO	71	11	0.777	0.644–0.910	0.397	4	76	0.65–0.845	64	0.354–0.848	93	29	2.09	0.947–4.62
**DN4-symptoms**	**168**	**57**	**0.713**	**0.634–0.791**	**0.369**	**3**	**70**	**0.629–0.766**	**67**	**0.537–0.775**	**86**	**43**	**2.11**	**1.441–3.082**
LBLP	74	28	0.716	0.606–0.826	0.348	3	72	0.605–0.806	61	0.424–0.764	83	45	1.82	1.126–2.953
					0.348	4	53	0.415–0.637	82	0.644–0.921	89	40	2.95	1.296–6.723
NSAP	23	18	0.661	0.484–0.837	0.374	3	65	0.449–0.812	72	0.491–0.875	75	62	2.35	1.052–5.238
PSNO	71	11	0.764	0.611–0.918	0.431	3	70	0.59–0.798	73	0.434–0.903	94	28	2.58	0.972–6.858
**DN4-signs A**	**168**	**49**	**0.781**	**0.709–0.852**	**0.537**	**1**	**76**	**0.692–0.82**	**78**	**0.641–0.87**	**92**	**49**	**3.39**	**2.003–5.75**
LBLP	73	26	0.744	0.637–0.850	0.479	1	67	0.557–0.768	81	0.621–0.942	91	47	3.49	1.562–7.799
NSAP	23	11	0.783	0.632–0.933	0.565	1	57	0.368–0.744	100	0.741–1.000	100	52	---	---
PSNO	72	12	0.763	0.608–0.917	0.417	1	92	0.83–0.961	50	0.254–0.746	92	50	1.83	1.037–3.242
**DN4-signs B**	**161**	**49**	**0.738**	**0.660–0.816**	**0.447**	**1**	**73**	**0.66–0.795**	**71**	**0.576–0.822**	**89**	**45**	**2.57**	**1.632–4.033**
LBLP	68	26	0.742	0.636–0.847	0.455	1	65	0.528–0.75	81	0.621–0.915	90	47	3.36	1.501–7.541
NSAP	21	11	0.777	0.606–0.948	0.576	1	67	0.454–0.828	91	0.623–0.984	93	59	7.33	1.104–48.691
PSNO	72	12	0.628	0.463–0.794	0.167	1	83	0.731–0.902	33	0.138–0.609	88	25	1.25	0.827–1.89
					0.167	2	67	0.552–0.765	50	0.254–0.746	89	20	1.33	0.74–2.403
**Grading A = B**														
**DN4 A**	138	81	0.771	0.709–0.833	0.396	4	75	0.676–0.818	64	0.533–0.738	78	60	2.10	1.549–2.861
**DN4 B**	135	75	0.744	0.673–0.814	0.382	4	76	0.677–0.82	63	0.514–0.727	7	59	2.02	1.487–2.755
**DN4-symptoms**	139	91	0.610	0.537–0.684	0.179	4	45	0.373–0.536	73	0.626–0.806	72	46	1.65	1.128–2.414
**DN4-signs A**	138	84	0.855	0.803–0.908	0.653	1	86	0.787–0.904	80	0.7–0.87	87	77	4.23	2.748–6.495
**DN4-signs B**	135	77	0.759	0.691–0.827	0.466	1	78	0.701–0.84	69	0.578–0.781	81	64	2.50	1.769–3.52

DN4: Douleur neuropathique en 4 questions; present NePC: Neuropathic pain component existing; Absent NePC: Neuropathic pain component not existing; AUC: Area under curve; 95%CI: 95% confidence interval; Sens.: Sensitivity; Spec.: Specificity; PPV: Positive predictive value; A: Physicians A; B: Physicians B; LBP: Patients suffering from low back and leg pain; NSAP: Patients suffering from neck shoulder arm pain; PSNO: Patients suffering from pain due to a suspected neuropathic origin.

In Table 6 and S2 Table we present the number of patients per group, values of the AUC, Youden index, cut-off score, true positives, false positives, false negatives, true negatives, sensitivity, specificity, positive and negative predictive values, positive and negative likelihood ratios, the diagnostic odds ratio, the a-priori chance for the existence (or not) of a NePC and false positive and negative ratios for all validity outcomes (DN4 A & B, DN4-symptoms, DN4-signs A & B) divided according to the pain classification and divided into LBLP, NSAP and PSNO (Table 6 and S2 Table).

### Test-retest reliability

Stability and responsiveness of the DN4-symptoms over time was assessed over a period of two weeks. The median sum score (IQR) of the DN4 at baseline for the total group was 3 (2–4), after two weeks it was 3 (2–4). Taking into consideration the fact that patients’ pain should not have changed (outcome based on the PGIC) because otherwise the ICC would not reflect the consistency of the DN4, test-retest reliability via ICC was 0.84 (excellent) (95%CI 0.80–0.87; n = 265). For the time gap of 7–21 days (to rule out the early or delayed return of questionnaires) between the first and second DN4-symptoms the ICC was 0.85 (excellent) (95% CI 0.79–0.90; n = 122). After three months, with no change in patients pain and a time gap of 60–120 days between the first and third DN4-symptoms, ICC was 0.79 (excellent) (95% CI 0.70–0.86; n = 102).

## Discussion

The DN4 seems, in this study, to help to identify a neuropathic pain component in a consecutive population of patients with chronic pain in a moderate way.

### Reliability

We used the concordant opinion about the classification of patients’ pain by two physicians as the gold standard. It is disputable if the term gold standard is practicable. However, as written by Versi[56] [57] “the gold standard is not the perfect test but merely the best available test…. Against which newer tests can be compared”. There are studies regarding the validity of the DN4 using only one physician’s opinion[21, 30]. To our opinion it is preferable to use two physicians as the gold standard, which is also performed in the original validation study of the DN4[17]. This might lead to less false positive or false negative outcomes which, of course, will lead to a more accurate validity outcome. The physicians in this study agreed on pain classification in 78% of the patients. In other studies without pre-stratification of patients on the target outcome the results for the physicians agreement were 53%[25] and 89%[27]. The kappa coefficient between the DN4 as filled in by physician A compared to the DN4 by physician B was ‘good’ with a high percentage of agreement. Test-retest reliability of the DN4-symptoms in this study was excellent. Based on these results DN4 seems to be reliable. However, it is possible that an instrument is reliable without being valid[58].

### Validity

To quantify the screening ability of the DN4, for the existence of a NePC, sensitivity and specificity can be used[59]. However, in clinical practice we want to know many how patients with a positive score on the DN4 really does have a NePC. To report this, the positive and negative predictive values are important because they give the proportion of patients with positive or negative test results which are correctly diagnosed[60]. The predictive value depends on the prevalence of NePC in the group of patients under study[60]. In our study the prevalence of NePC was high, 75%. The higher the prevalence of NePC in the group under study the more sure it is that a positive outcome of the DN4 indicates the presence of a NePC, but the less sure it is that a negative DN4 outcome indicates absent NePC[60]. The likelihood ratio gives an indication of the value of the DN4 for increasing certainty about a positive diagnosis[60]. A higher likelihood ratio might indicate that the DN4 is useful, but is still not sure that a positive outcome of the DN4 is a good indicator for the presence of a NePC[60]. In the literature there are, as far as we know, no ‘cut-off’ scores for the validity indices. In our study we found a sensitivity of 75% (DN4-symptoms 70%), a specificity of 76% (DN4-symptoms 67%), positive predictive value of 92%, negative predictive value of 46% and the positive and negative likelihood ratios were respectively 3.09 and 0.34. In the original study by Bouhassira et al.[17] patients with only ‘typical’ neuropathic or nociceptive entities and a VAS of ≥40 mm (0–100mm) were included. They found a sensitivity of 83% and a specificity of 90%. For the DN4-symptoms the sensitivity was 78% and the specificity 81%. The Dutch version of the DN4[20] was validated before in a consecutive group of patients suffering from chronic pain for more than three months with a pain score of 5 or higher on a 0–10 numeric rating scale (NRS)[25]. For the DN4 a sensitivity of 75% and a specificity of 79% was found. For the DN4-symptoms version sensitivity was 74% and the specificity 79%. Van Seventer et al. concluded that the DN4 was a diagnostic tool with a good ability to discriminate between neuropathic pain and nociceptive pain[25]. However, the paper by Bouhassira et al.[17] and the paper by Van seventer et al.[25] both didn’t report the predictive values and likelihood ratios. Inappropriate screening might result in higher health care costs due to more diagnostic testing or even lead to a harmful treatment for the patient[61]. It seems that the validity indices in our study are resulting in a lower score for the DN4 as in the original publication[17] and than in other studies[4, 21, 23–28, 30, 31, 62–67]. This might have several reasons. At first, we did not pre-stratify on the target outcome. In studies, besides the original validation study[17] with pre-stratification on the target outcome[23, 24, 26, 28, 31] (neuropathic or non-neuropathic pain), the sensitivity of the DN4 was ranging from 90%[26] till 100%[24], the specificity from 93%[24]-97%[23, 28]. In studies where there was no pre-stratification on the target outcome (neuropathic or non-neuropathic pain), the sensitivity of the DN4 was ranging from 80%[21] till 100%[30], the specificity ranges from 78%[21, 27] till 87%[30]. These results are showing that the validity of the DN4 is lower in studies without pre-stratification than in studies were patients were stratified based on their pain classification before entering the study. In studies with specified diseases as spinal cord injury[64]; diabetes[63, 64]; leprosy[65, 66]; FBSS[67], chronic low back pain[4] and in patients with cancer before starting with chemotherapy[68], the sensitivity (62%-100%) and specificity (44%-93%) ranges were much wider. Our results, also when separated into results for LBLP, NSAP and PSNO, falls within these ranges. This indicates that the neuropathic pain component is not always clear and/or easy to classify by use of the DN4 in the different medical conditions. Secondly, in our study we did not have a minimum level of pain as an inclusion criteria. In seven studies a minimal level of pain (on a rating scale of 0–10) was not an inclusion criteria [21, 23, 31, 62, 63, 65, 66]. In other studies a level ≥ three[64, 67], ≥ four[4, 17, 24, 26, 28, 30] or ≥ five[25, 27] is set as an inclusion criterium. As shown by Perez et al[21], pain severity has a major influence on the sensitivity and specificity of the DN4. A severity of < 40 mm on a 0-100mm VAS resulted in a sensitivity of 56% and a specificity of 67%. For moderate pain (between 40mm en 70mm on a 0-100mm VAS) it was 85% and 84% respectively, and >70 mm sensitivity was 80% and specificity was 74%[21]. In a study by Marksman[67] in patients after FBSS it was showed that the presence of neuropathic characteristics, as determined by the DN4, was associated with a higher pain intensity. These facts are crucial for the validation of a screening instrument because such a tool must be valid for use in daily clinical out-patient practice and/or for epidemiological purposes.

As a second comparison, we validated the DN4 in comparison with the grading system[32] [21]. In this study, we combined ‘unlikely’ and possible neuropathic pain as absent NePC and probable and definite as present NePC, which resulted in an average sensitivity for the DN4 of 76% and an average specificity of 64%. In patients with a failed back surgery syndrome[67], the validation of the DN4 resulted in a sensitivity of 62% and a specificity of 44%. In a study by Sadler et al [69] where patients with neuropathic pain were compared to musculoskeletal pain the sensitivity was 76% and the specificity was 70%. However, in patients with a more mixed pain the sensitivity and specificity descended to 59% and 70% respectively. Abdallah et al [36] compared the DN4 with the grading system in patients after breast tumor resection with and without paravertebral blocks. This resulted in a sensitivity of 90% and a specificity of 60% to identify patients with chronic neuropathic pain based on the outcome of the grading system. However, this outcome was not validated by (expert) physicians. The distinction between possible neuropathic pain and probable or definite neuropathic pain is of high importance because the outcome forms the basis for selecting a different treatment strategy[34]. The combination of outcomes in our study might have resulted in a lower sensitivity and a bit higher specificity in comparison with the classification in the study of Abdallah et al[36].

#### Douleur Neuropathique en 4 questions (DN4)

Bouhassira [17] presented the DN4 as a clinician-administered questionnaire. In different studies not a physician but a research coordinator[30], a nurse[25] or the patient self [25, 70] filled in the DN4. In our study we gave the patient the questionnaire with the 7-items (DN4-symptoms) to fill them in after the physical examinations. The three examination-items (DN4-signs) were incorporated in the standardized assessment form which should be filled in by the physician. We presented the DN4 total sum score as well as the DN4-signs score separately for physicians A and B. This is due to the fact that it is only possible to have one outcome when the sign-items were performed by one physician.

### Strength and weaknesses

There are several strengths in this study. At first, this study reflects daily clinical practice. In this study, we included a large cohort of patients irrespective of the predominant origin of the pain and level of pain which corresponds to a typical daily clinical patient population. These patients were associated with the most common specified medical conditions for pain (i.e. LBLP or NSAP or PSNO) and classified by two, independently working, physicians. Moreover, patients were referred from primary care to secondary and tertiary pain clinics and were assessed for their complaints for the first time at the time of inclusion in this study. This limits the risk of systematic bias and also reflects daily clinical practice. Secondly, we used a standardized assessment form in which the bedside examination and the grading system[32, 33] and the DN4-signs were incorporated. This might, however, have led to an influence on each other which made the physician more sure about the final classification of patients pain and thus made the gold standard stronger. There are also some weaknesses in this study. As said before, we have not used the DN4-symptoms as a interview by a physician but as a questionnaire which has to be filled in by the patient. This might have had an influence on the reliability and validity. In the revised EFNS guidelines on neuropathic pain assessment[42] it is suggested that “The seven sensory descriptors can be used as a self-report questionnaire with similar results”. Moreover, above the official Dutch version[20, 25] of the DN4 is written in Dutch: “To be completed by the patient”. In the paper by van Seventer et al the agreement between the patient administered and a nurse administered was good till very good for the first seven items[25]. It would be of interest to see if there are differences in the outcome when the DN4 is filled in by the patient himself or as an interview by the pain physician. Questions by the patient to the nurse of via telephone to the researcher regarding the DN4 were very rare. However, we didn’t keep track of the questions. Another limitation is the fact that we only tested the test-retest reliability regarding the DN4-symptoms and not the DN4-signs to prevent the patient to come back to the hospital only for these test-items. Another weakness is the gold standard which is, for now, the best measure for the existence of a neuropathic pain component but the result is still open for discussion.

### Suggestions for the validation of neuropathic pain screening tools

Validation of screening tools should be performed in a standardized manner and described in detail, but performed in a setting which is comparable to a daily clinical practice. A research setting might be different from a clinical setting and thus might have influence on the patient and on the study results. The group of patients as well as the physicians under study should be comparable to the patients/physicians for who the tool is intended. Pre-stratification on the target outcome must be avoided (especially the exclusion of the so called mixed pain), because this will lead to a non-clinical situation and thus decreases the validity and generalizability of the instrument[16, 71].

### Conclusion

The validity of DN4-signs is equal to the DN4 outcome and, importantly, both are more valid than the DN4-symptoms alone. It seems that the patients’ symptoms and signs doesn’t reliably reflect the underlying mechanisms, indicating there is a need for a more objective way to assess patients’ pain to facilitate improvement in the treatment of patients with pain. The physicians’ assessment cannot be replaced by a screening tool as the DN4, but gives the physician a little hint towards the (non-)existence of neuropathic pain component.

## Supporting information

S1 Table Loading factors of the three versions of the DN4 according to the rotated component matrix factor analysis. (DOCX)

S2 Table The area under the curve and the sensitivity / specificity at the optimal cut-off point of the DN4 under the condition of equal costs of misclassification to classify a neuropathic pain component by the classification and the grading system of the physicians. PD-Q: PainDETECT questionnaire; Present NePC: Neuropathic pain component existing; Absent NePC: Neuropathic pain component not existing; AUC: Area under curve; Std.Error: Standard error; Asymp. Sig.: Asymptotic Significance; 95%CI: 95% confidence interval; Sens.: Sensitivity; Spec.: Specificity; +DV: Positive diagnostic value; -DV: Negative diagnostic value; +LR: Positive likelihood ratio; -LR: Negative likelihood ratio; P[Z+]: a-priori chance for the existence of a NePC; P[Z-]: a-priori chance for no existence of NePC; FPR: False positive ratio; FNR: False negative ratio; A: Physician A; B: Physician B; LBLP: Low back and leg pain; NSAP: Neck shoulder arm pain; PSNO: Pain of suspected neuropathic origin.
(DOCX)
