# Supplementary material for: Investigating the validity of the DN4 in a consecutive population of patients with chronic pain
Source: PLoS One. 2017 Nov 30;12(11):e0187961. doi: 10.1371/journal.pone.0187961 (PMC5708633; doi:10.1371/journal.pone.0187961)
Supplement: S1 Table — (DOCX) [file pone.0187961.s001.docx]

**Supplement Table S1:** **Loading factors of the three versions of the DN4 according to the rotated component matrix factor analysis**

| DN4 | Component (Physicians A) | | | |  | Component (Physicians B) | | |
| --- | --- | --- | --- | --- | --- | --- | --- | --- |
|  | 1 | 2 | 3 | 4 |  | 1 | 2 | 3 |
| Burning | 0.251 | 0.279 | **0.292** | 0.149 |  |  | **0.542** |  |
| Painful cold |  | **0.616** |  |  |  |  | **0.497** |  |
| Electric shocks |  |  | **0.719** |  |  |  | 0.287 | **0.649** |
| Tingling |  | **0.676** |  |  |  |  | **0.425** | 0.418 |
| Pins and needles |  | 0.353 | **0.445** | 0.271 |  |  | **0.636** |  |
| Numbness |  |  | **0.709** | --- |  | 0.262 | **0.602** |  |
| Itching |  |  |  | **0.857** |  |  | **0.484** |  |
| Hypoesthesia to touch | **0.872** |  |  |  |  | **0.905** |  |  |
| Hypoesthesia to prick | 0.375 | **0.630** |  |  |  | 0.297 |  | **0.579** |
| Brushing | **0.896** |  |  |  |  | **0.907** |  |  |
| *Cronbach’s alpha* | *0.809* | *0.370* | *0.509* |  |  | *0.836* | *0.482* | *0.236* |

Loading factors < 0.25 are omitted to improve readability

| DN4-symptoms | Component | |
| --- | --- | --- |
|  | 1 | 2 |
| Burning | **0.439** |  |
| Painful cold | **0.515** |  |
| Electric shocks |  | **0.715** |
| Tingling |  | **0.654** |
| Pins and needles | **0.529** | 0.347 |
| Numbness | **0.625** |  |
| Itching | **0.684** |  |
| *Cronbach’s alpha* | *0.476* | *0.368* |

Loading factors < 0.25 are omitted to improve readability

| DN4-signs | Component (Physicians A) |  | Component (Physicians B) |
| --- | --- | --- | --- |
|  | 1 |  | 1 |
| Hypoesthesia to touch | **0.881** |  | **0.912** |
| Hyopoesthesia to prick | **0.515** |  | **0.405** |
| Brushing | **0.880** |  | **0.895** |
| *Cronbach’s alpha* | *0.675* |  | *0.663* |

DN4-examination via component matrix; One component extracted, the solution couldn’t be rotated.
