# Supplementary material for: Investigating the validity of the DN4 in a consecutive population of patients with chronic pain
Source: PLoS One. 2017 Nov 30;12(11):e0187961. doi: 10.1371/journal.pone.0187961 (PMC5708633; doi:10.1371/journal.pone.0187961)
Supplement: S2 Table — PD-Q: PainDETECT questionnaire; Present NePC: Neuropathic pain component existing; Absent NePC: Neuropathic pain component not existing; AUC: Area under curve; Std.Error: Standard error; Asymp. Sig.: Asymptotic Significance; 95%CI: 95% confidence interval; Sens.: Sensitivity; Spec.: Specificity; +DV: Positive diagnostic value; -DV: Negative diagnostic value; +LR: Positive likelihood ratio; -LR: Negative likelihood ratio; P[Z+]: a-priori chance for the existence of a NePC; P[Z-]: a-priori chance for no existence of NePC; FPR: False positive ratio; FNR: False negative ratio; A: Physician A; B: Physician B; LBLP: Low back and leg pain; NSAP: Neck shoulder arm pain; PSNO: Pain of suspected neuropathic origin. (DOCX) [file pone.0187961.s002.docx]

**Supplement Table S2:**

|  | **Present NePC** | **Absent NePC** | **AUC** | **Std. Error** | **Asymp. Sig.** | **95%CI** | **YI** | **Cut-off** | **T+** | **F+** | **F-** | **T-** | **Sens %** | **Spec %** | **+PV %** | **-PV %** | **+LR** | **-LR** | **P[Z+]** | **P[Z-]** | **FPR %** | **FNR %** |
| --- | --- | --- | --- | --- | --- | --- | --- | --- | --- | --- | --- | --- | --- | --- | --- | --- | --- | --- | --- | --- | --- | --- |
| **Classification**  **A vs DN4-7**  LBLP (n)  NSAP (n)  PSNO (n)  **Classification**  **B vs DN4-7**  LBLP (n)  NSAP (n)  PSNO (n)  **Classification**  **A=B vs DN4-7**  LBLP (n)  NSAP (n)  PSNO (n)  **Classification**  **A vs DN4-10A**  LBLP (n)  NSAP (n)  PSNO (n)  **Classification**  **B vs DN4-10B**  LBLP (n)  NSAP (n)  PSNO (n)  **Classification**  **A=B vs DN4-10A**  LBLP (n)  NSAP (n)  PSNO (n)  **Classification**  **A=B vs DN4-10B**  LBLP (n)  NSAP (n)  PSNO (n) | **206**  94  28  84  **193**  83  28  82  **168**  74  23  71  **203**  91  28  84  **184**  76  26  82  **166**  72  23  71  **159**  67  21  71 | **82**  37  23  22  **95**  48  23  24  **57**  28  18  11  **72**  35  15  22  **79**  42  13  24  **47**  26  10  11  **48**  26  11  11 | **0.693**  0.689  0.658  0.726  **0.645**  0.684  0.586  0.611  **0.713**  0.716  0.661  0.764  **0.766**  0.775  0.726  0.777  **0.736**  0.759  0.664  0.683  **0.829**  0.823  0.763  0.836  **0.807**  0.821  0.725  0.777 | **0.035**  0.050  0.081  0.060  **0.034**  0.047  0.081  0.065  **0.040**  0.056  0.090  0.078  **0.030**  0.042  0.088  0.052  **0.032**  0.043  0.094  0.061  **0.031**  0.043  0.095  0.063  **0.033**  0.043  0.100  0.068 | **0.000**  0.001  0.054  0.001  **0.000**  0.000  0.293  0.098  **0.000**  0.001  0.081  0.005  **0.000**  0.000  0.015  0.000  **0.000**  0.000  0.098  0.007  **0.000**  0.000  0.018  0.000  **0.000**  0.000  0.039  0.003 | **0.625-0.761**  0.591-0.788  0.499-0.818  0.608-0.843  **0.578-0.711**  0.592-0.777  0.427-0.746  0.484-0.739  **0.634-0.791**  0.606-0.826  0.484-0.837  0.611-0.918  **0.717-0.836**  0.692-0.859  0.554-0.898  0.675-0.879  **0.673-0.798**  0.674-0.844  0.481-0.848  0.563-0.802  **0.767-0.890**  0.738-0.908  0.576-0.950  0.713-0.959  **0.742-0.872**  0.736-0.906  0.529-0.921  0.644-0.910 | **0.338**  0.298  0.366  0.384  **0.245**  0.348  0.208  0.159  **0.369**  0.348  0.374  0.431  **0.394**  0.448  0.395  0.459  **0.365**  0.470  0.269  0.258  **0.513**  0.544  0.439  0.543  **0.498**  0.554  0.442  0.397 | 3  3  3  3  **4**  4  3  3  **3**  3  4  3  3  **4**  4  2  5  **4**  4  3  4  6  **4**  4  3  5  **4**  4  4  4 | **140**  61  20  59  **90**  41  18  54  **118**  53  39  15  50  **142**  59  26  50  **130**  52  19  17  28  **124**  53  17  45  **119**  50  15  54 | **28**  13  8  7  **21**  7  10  12  **19**  11  5  5  3  **22**  7  8  3  **27**  9  6  5  2  **11**  5  3  1  **12**  5  3  4 | **66**  33  8  25  **103**  42  10  28  **50**  21  35  8  21  **61**  32  2  34  **54**  24  7  9  54  **42**  19  6  26  **40**  17  6  17 | **54**  24  15  15  **74**  41  13  12  **38**  17  23  13  8  **50**  28  7  19  **52**  33  7  8  22  **36**  21  7  10  **36**  21  8  7 | **68**  65  71  70  **47**  49  64  66  **70**  72  53  65  70  **70**  65  93  60  **71**  68  73  65  34  **75**  74  74  63  **75**  75  71  76 | **66**  65  65  68  **78**  85  57  50  **67**  61  82  72  73  **69**  80  47  86  **66**  79  54  62  92  **77**  81  70  91  **75**  81  73  64 | **83**  82  71  89  **81**  85  64  82  **86**  83  89  75  94  **87**  89  76  94  **83**  85  76  77  93  **92**  91  85  98  **91**  91  83  93 | **45**  42  65  38  **42**  49  57  30  **43**  45  40  62  28  **45**  47  78  36  **49**  58  50  47  29  **46**  53  54  28  **47**  55  57  29 | **1.99**  1.85  2.05  2.21  **2.11**  3.39  1.48  1.32  **2.11**  1.82  2.95  2.35  2.58  **2.29**  3.24  1.74  4.37  **2.07**  3.19  1.58  1.70  4.10  **3.19**  3.83  2.46  6.97  **2.99**  3.88  2.62  2.09 | **0.49**  0.54  0.44  0.44  **0.69**  0.59  0.63  0.68  **0.45**  0.47  0.58  0.48  0.41  **0.43**  0.44  0.15  0.47  **0.45**  0.40  0.50  0.56  0.72  **0.33**  0.33  0.37  0.40  **0.34**  0.31  0.39  0.38 | **72**  72  55  79  **67**  63  55  77  **75**  73  73  56  87  **74**  72  65  79  **70**  64  67  67  77  **78**  73  70  87  **77**  72  66  87 | **28**  28  45  21  **33**  37  45  23  **25**  27  27  44  13  **26**  28  35  21  **30**  36  33  33  23  **22**  27  30  13  **23**  28  34  13 | **34**  35  35  32  **22**  15  43  50  **33**  39  18  28  27  **31**  20  53  14  **34**  21  46  38  8  **23**  19  30  9  **25**  19  27  36 | **32**  35  29  30  **53**  51  36  34  **30**  28  47  35  30  **30**  35  7  40  **29**  32  27  35  66  **25**  26  26  37  **25**  25  29  24 |
|  | **Present NePC** | **Absent NePC** | **AUC** | **Std. Error** | **Asymp. Sig.** | **95%CI** | **Youden**  **index** | **Cut-off** | **T+** | **F+** | **F-** | **T-** | **Sens %** | **Spec %** | **+PV %** | **-PV %** | **+LR** | **-LR** | **P[Z+]** | **P[Z-]** | **FPR %** | **FNR %** |
| **Grading**  **A vs DN4-7**  LBLP (n)  NSAP (n)  PSNO (n)  **Grading**  **B vs DN4-7**  LBLP (n)  NSAP (n)  PSNO (n)  **Grading**  **A=B vs DN4-7**  LBLP (n)  NSAP (n)  PSNO (n)  **Grading**  **A vs DN4-10A**  LBLP (n)  NSAP (n)  PSNO (n)  **Grading B vs DN4-10 B**  LBLP (n)  NSAP (n)  PSNO (n)  **Grading A=B vs DN4-10A**  LBLP (n)  NSAP (n)  PSNO (n)  **Grading A=B vs DN4-10B**  LBLP (n)  NSAP (n)  PSNO (n) | **171**  61  17  93  **158**  56  19  83  **139**  48  13  78  **170**  60  17  93  **153**  52  18  83  **138**  47  13  78  **135**  45  12  78 | **112**  69  30  13  **127**  74  32  21  **91**  60  24  7  **102**  65  24  13  **107**  65  21  21  **81**  56  18  7  **75**  53  15  7 | **0.608**  0.620  0.558  0.690  **0.572**  0.612  0.484  0.562  **0.610**  0.638  0.513  0.730  **0.741**  0.728  0.643  0.772  **0.708**  0.723  0.545  0.701  **0.771**  0.754  0.641  0.877  **0.744**  0.754  0.550  0.761 | **0.034**  0.049  0.087  0.073  **0.034**  0.050  0.089  0.070  **0.038**  0.054  0.102  0.077  **0.030**  0.045  0.086  0.072  **0.033**  0.049  0.095  0.071  **0.032**  0.049  0.101  0.068  **0.036**  0.050  0.113  0.103 | **0.002**  0.019  0.514  0.027  **0.038**  0.029  0.846  0.380  **0.005**  0.014  0.899  0.045  **0.000**  0.000  0.122  0.002  **0.000**  0.000  0.632  0.005  **0.000**  0.000  0.186  0.001  **0.000**  0.000  0.661  0.023 | **0.541-0.675**  0.523-0.717  0.388-0.728  0.547-0.833  **0.505-0.638**  0.513-0.711  0.309-0.658  0.425-0.699  **0.537-0.684**  0.531-0.744  0.314-0.712  0.580-0.880  **0.681-0.800**  0.639-0.817  0.475-0.812  0.631-0.914  **0.644-0.772**  0.628-0.819  0.358-0.732  0.562-0.840  **0.709-0.833**  0.658-0.850  0.444-0.838  0.745-1.000  **0.673-0.814**  0.655-0.852  0.329-0.771  0.558-0.964 | **0.179**  0.215  0.149  0.375  **0.127**  0.198  0.054  0.115  **0.179**  0.250  0.074  0.436  **0.357**  0.346  0.248  0.445  **0.321**  0.377  0.190  0.356  **0.396**  0.393  0.269  0.626  **0.382**  0.413  0.217  0.507 | **4**  4  2  4  **3**  4  5  3  **4**  4  3  4  **4**  4  4  4  **4**  4  4  3  **4**  6  4  4  **4**  4  4  3 | **78**  29  15  42  **101**  27  4  53  **63**  24  8  34  **124**  42  12  70  **112**  38  12  77  **104**  21  10  60  **102**  33  9  73 | **31**  18  22  1  **65**  21  5  11  **25**  15  13  0  **38**  23  11  4  **44**  23  10  12  **29**  3  9  1  **28**  17  8  3 | **93**  32  2  51  **57**  29  15  30  **76**  24  5  44  **46**  18  5  23  **41**  14  6  6  **34**  26  3  18  **33**  12  3  5 | **81**  51  8  12  **62**  53  27  10  **66**  45  11  7  **64**  42  13  9  **63**  42  11  9  **52**  53  9  6  **47**  36  7  4 | **46**  48  88  45  **64**  48  21  64  **45**  50  62  44  **73**  70  71  75  **73**  73  67  93  **75**  45  77  77  **76**  73  75  94 | **72**  74  27  92  **49**  72  84  48  **73**  75  46  100  **63**  65  54  69  **59**  65  52  43  **64**  95  50  86  **63**  68  47  57 | **72**  62  41  98  **61**  56  44  83  **72**  62  38  100  **72**  65  52  95  **72**  62  55  87  **78**  88  53  98  **78**  66  53  96 | **47**  61  80  19  **52**  65  64  25  **46**  65  69  14  **58**  70  72  28  **61**  75  65  60  **60**  67  75  25  **59**  75  70  44 | **1.65**  1.82  1.20  5.87  **1.25**  1.70  1.35  1.22  **1.65**  2.00  1.14  **1.96**  1.98  1.54  2.45  **1.78**  2.07  1.40  1.62  **2.10**  8.34  1.54  5.38  **2.02**  2.29  1.41  2.18 | **0.75**  0.71  0.44  0.59  **0.74**  0.72  0.94  0.76  **0.75**  0.67  0.84  0.56  **0.43**  0.46  0.54  0.36  **0.46**  0.42  0.64  0.17  **0.38**  0.58  0.46  0.27  **0.39**  0.39  0.54  0.11 | **60**  47  36  88  **55**  43  37  80  **60**  44  35  92  **63**  48  41  88  **59**  44  46  80  **63**  46  42  92  **64**  46  44  92 | **40**  53  64  12  **45**  57  63  20  **40**  56  65  8  **38**  52  59  12  **41**  56  54  20  **37**  54  58  8  **36**  54  56  8 | **28**  26  73  8  **51**  28  16  52  **27**  25  54  0  **37**  35  46  31  **41**  35  48  57  **36**  5  50  14  **37**  32  53  43 | **54**  52  12  55  **36**  52  79  36  **55**  50  38  56  **27**  30  29  25  **27**  27  33  7  **25**  55  23  23  **24**  27  25  6 |
|  | **Present NePC** | **Absent NePC** | **AUC** | **Std. Error** | **Asymp. Sig.** | **95%CI** | **Youden**  **index** | **Cut-off** | **T+** | **F+** | **F-** | **T-** | **Sens %** | **Spec %** | **+PV %** | **-PV %** | **+LR** | **-LR** | **P[Z+]** | **P[Z-]** | **FPR %** | **FNR %** |
| **Classification A = Grading A**  **Vs DN4-7**  LBLP (n)  NSAP (n)  PSNO (n)  **Classification A = Grading A**  **Vs DN4-10A**  LBLP (n)  NSAP (n)  PSNO (n)  **Classification B = Grading B**  **Vs DN4-7**  LBLP (n)  NSAP (n)  PSNO (n)  **Classification B = Grading B**  **Vs DN4-10B**  LBLP (n)  NSAP (n)  PSNO (n) | **154**  59  15  80  **153**  58  15  80  **139**  50  16  73  **135**  47  15  73 | **62**  35  18  9  **54**  33  12  9  **76**  42  20  14  **61**  37  10  14 | **0.691**  0.698  0.613  0.801  **0.831**  0.813  0.725  0.876  **0.636**  0.704  0.559  0.600  **0.787**  0.838  0.637  0.728 | **0.040**  0.055  0.101  0.063  **0.030**  0.044  0.105  0.048  **0.039**  0.054  0.100  0.084  **0.034**  0.044  0.114  0.084 | **0.000**  0.001  0.270  0.003  **0.000**  0.000  0.048  0.000  **0.001**  0.001  0.545  0.239  **0.000**  0.000  0.255  0.007 | **0.612-0.770**  0.590-0.805  0.415-0.811  0.677-0.924  **0.772-0.890**  0.726-0.900  0.519-0.931  0.782-0.969  **0.560-0.713**  0.597-0.811  0.364-0.755  0.436-0.764  **0.720-0.854**  0.751-0.924  0.413-0.861  0.564-0.893 | **0.311**  0.292  0.278  0.418  **0.499**  0.512  0.433  0.600  **0.232**  0.373  0.188  0.158  **0.441**  0.565  0.300  0.418 | **3**  4  3  2  **4**  4  2  5  **4**  4  3  3  **4**  4  4  3 | **105**  29  10  69  **116**  42  14  48  **67**  27  11  48  **106**  38  12  67 | **23**  7  7  4  **14**  7  6  0  **19**  7  10  7  **21**  9  5  7 | **49**  30  5  11  **37**  16  1  32  **72**  23  5  25  **29**  9  3  6 | **39**  28  11  5  **40**  26  6  9  **57**  35  10  7  **40**  28  5  7 | **68**  49  67  86  **76**  72  93  60  **48**  54  69  66  **79**  81  80  92 | **63**  80  61  56  **74**  79  50  100  **75**  83  50  50  **66**  76  50  50 | **82**  81  59  95  **89**  86  70  100  **78**  79  52  87  **83**  81  71  91 | **44**  48  69  31  **52**  62  86  22  **44**  60  67  22  **58**  76  63  54 | **1.84**  2.46  1.71  1.94  **2.92**  3.41  1.87  **1.93**  3.24  1.38  1.32  **2.28**  3.32  1.60  1.84 | **0.51**  0.64  0.55  0.25  **0.33**  0.35  0.13  0.40  **0.69**  0.55  0.63  0.68  **0.33**  0.25  0.40  0.16 | **71**  63  45  90  **74**  64  56  90  **65**  54  44  0.84  **69**  56  60  84 | **29**  37  55  10  **26**  36  44  10  **35**  46  56  16  **31**  44  40  16 | **37**  20  39  44  **26**  21  50  0  **25**  17  50  50  **34**  24  50  50 | **32**  51  33  14  **24**  28  7  40  **52**  46  31  34  **21**  19  20  8 |
|  | **Present NePC** | **Absent NePC** | **AUC** | **Std. Error** | **Asymp. Sig.** | **95%CI** | **Youden**  **index** | **Cut-off** | **T+** | **F+** | **F-** | **T-** | **Sens %** | **Spec %** | **+PV %** | **-PV %** | **+LR** | **-LR** | **P[Z+]** | **P[Z-]** | **FPR %** | **FNR %** |
| **Classification A = Grading A = Classification B = Grading B vs DN4-7**  LBLP (n)  NSAP (n)  PSNO (n)  **Classification A = Grading A = Classification B = Grading B vs DN4-10 A**  LBLP (n)  NSAP (n)  PSNO (n)  **Classification A = Grading A = Classification B = Grading B vs DN4-10 B**  LBLP (n)  NSAP (n)  PSNO (n) | **118**  43  12  63  **117**  42  12  63  **114**  40  11  63 | **42**  26  12  4  **34**  24  6  4  **34**  24  6  4 | **0.670**  0.721  0.549  0.750  **0.859**  0.857  0.667  0.937  **0.822**  0.878  0.561  0.758 | **0.048**  0.063  0.123  0.095  **0.033**  0.045  0.143  0.043  **0.040**  0.042  0.159  0.128 | **0.001**  0.002  0.686  0.095  **0.000**  0.000  0.261  0.004  **0.000**  0.000  0.688  0.085 | **0.576-0.765**  0.598-0.844  0.307-0.791  0.563-0.937  **0.795-0.923**  0.769-0.946  0.386-0.947  0.853-1.000  **0.745-0.900**  0.795-0.961  0.250-0.872  0.506-1.000 | 0.286  0.366  0.250  0.476  0.551  0.577  0.333  0.778  0.504  0.617  0.318  0.500 | 4  4  3  4  4  4  3  4  4  4  4  2 | **59**  24  8  30  **92**  33  10  49  **91**  33  9  63 | **9**  5  5  0  **8**  5  3  0  **10**  5  3  2 | **59**  19  4  33  **25**  9  2  14  **23**  7  2  0 | **33**  21  7  4  **26**  19  3  4  **24**  19  3  2 | **50**  56  67  48  **79**  79  83  78  **80**  83  82  100 | **79**  81  58  100  **76**  79  50  100  **71**  79  50  50 | **87**  83  62  100  **92**  87  77  100  **90**  87  75  97 | **36**  53  64  11  **51**  68  60  22  **51**  73  60  100 | **2.33**  2.90  1.60  **3.34**  3.77  1.67  **2.71**  3.96  1.64  2.00 | **0.64**  0.55  0.57  0.52  **0.28**  0.27  0.33  0.22  **0.29**  0.22  0.36  0.00 | **0.74**  0.62  0.50  0.94  **0.77**  0.64  0.67  0.94  **0.77**  0.63  0.65  0.94 | **0.26**  0.38  0.50  0.06  **0.23**  0.36  0.33  0.06  **0.23**  0.38  0.35  0.06 | **0.21**  0.19  0.42  0.00  **0.24**  0.21  0.50  0.00  **0.29**  0.21  0.50  0.50 | **0.50**  0.44  0.33  0.52  **0.21**  0.21  0.17  0.22  **0.20**  0.18  0.18  0.00 |

*PD-Q: PainDETECT questionnaire; Present NEPC: Neuropathic pain component existing; Absent NePC: Neuropathic pain component not existing; AUC: Area under curve; Std.Error: Standard error; Asymp. Sig.: Asymptotic Significance; 95%CI: 95% confidence interval; Sens.: Sensitivity; Spec.: Specificity; +DV: Positive diagnostic value; -DV: Negative diagnostic value; +LR: Positive likelihood ratio; -LR: Negative likelihood ratio; P[Z+]: a-priori chance for the existence of a NePC; P[Z-]: a-priori chance for no existence of NePC; FPR: False positive ratio; FNR: False negative ratio; A: Physician A; B: Physician B; LBLP: Low back and leg pain; NSAP: Neck shoulder arm pain; PSNO: Pain of suspected neuropathic origin*
